# Intercellular adhesion molecule 1 rs5498 polymorphism is associated with the risk of myocardial infarction

**DOI:** 10.18632/oncotarget.17529

**Published:** 2017-04-29

**Authors:** Pengfei Hu, Tao Dai, Weiwei Yu, Ying Luo, Shuwei Huang

**Affiliations:** ^1^ Department of Cardiology, The Second Affiliated Hospital of Zhejiang Chinese Medical University, Hangzhou, Zhejiang, China; ^2^ Department of Cardiology, Yuhuangding Hospital Affiliated to Qingdao University, Yantai, Shandong, China

**Keywords:** ICAM-1, rs5498, myocardial infarction, meta-analysis

## Abstract

Several studies addressed the association between Intercellular adhesion molecule 1 (ICAM-1) rs5498 polymorphism and Myocardial Infarction (MI) risk. However, they addressed conflicting findings. Therefore, the aim of this study was to explore whether ICAM-1 gene rs5498 polymorphism plays an important role in modifying the risk of MI. A meta-analysis was conducted on the association between ICAM-1 rs5498 polymorphism and MI. 12 eligible studies involving 1,696 cases and 3,039 controls were included in the meta-analysis. Meta-analysis revealed that ICAM-1 rs5498 polymorphism showed a strongly positive correlation with MI and could be viewed as a protective factor for MI. Furthermore, subgroup analysis according to ethnicity indicated that ICAM-1 rs5498 polymorphism decreased the risk of MI among Caucasian and Asian populations. In conclusion, ICAM-1 rs5498 polymorphism was associated with the decreased risk of MI. Larger sample size studies with more diverse ethnic populations are needed to confirm these findings.

## INTRODUCTION

Cardiovascular disease (CVD), including myocardial infarction (MI), is the number one cause of death [1]. Suspected MI is a common reason for emergency hospital attendance and admission [2]. It has been suggested that biomarkers of inflammation, such as increased blood homocysteine [3], C-reactive protein [3], and cytokine levels [4], may be new risk factors for CVD. However, these biomarkers are poorly specific and susceptible. Inherited gene variants are less influenced by environment factors, and may be a better marker of individual MI susceptibility [5].

Intercellular adhesion molecule 1 (ICAM-1) is the member of the adhesion immunoglobulin super family that maps to chromosome 19 p13.2-p13.3 codes for 505 amino acids with five extracellular domains [6]. ICAM-1 is implicated in neutrophil and monocyte-endothelial cell adhesion, processes contributing to myocardial neutrophil infiltration and microvascular coronary slow flow, both viewed as important to the pathophysiologic responses in acute myocardial infarction (AMI) [7]. Patients with stable angina pectoris who developed MI had elevated serum levels of soluble ICAM-1, indicating increased inflammatory activity [8]. Kamijikkoku *et al*. found that a persistent increase in plasma soluble ICAM-1 levels may indirectly implicate vascular inflammation, which could predict the risk of early coronary restenosis after emergency angioplasty in patients with AMI [9]. Moreover, Niessen *et al*. identified that inhibition of ICAM-1 expression in the heart dramatically reduces infarct size [10].

Recently, several studies addressed the association between ICAM-1 gene rs5498 polymorphism and MI risk. However, they yielded contradictory and inconclusive findings [11–22]. Thus, we undertook this meta-analysis to evaluate whether ICAM-1 rs5498 polymorphism is associated with MI risk.

## RESULTS

### Characteristics of the included studies

**Figure 1:**
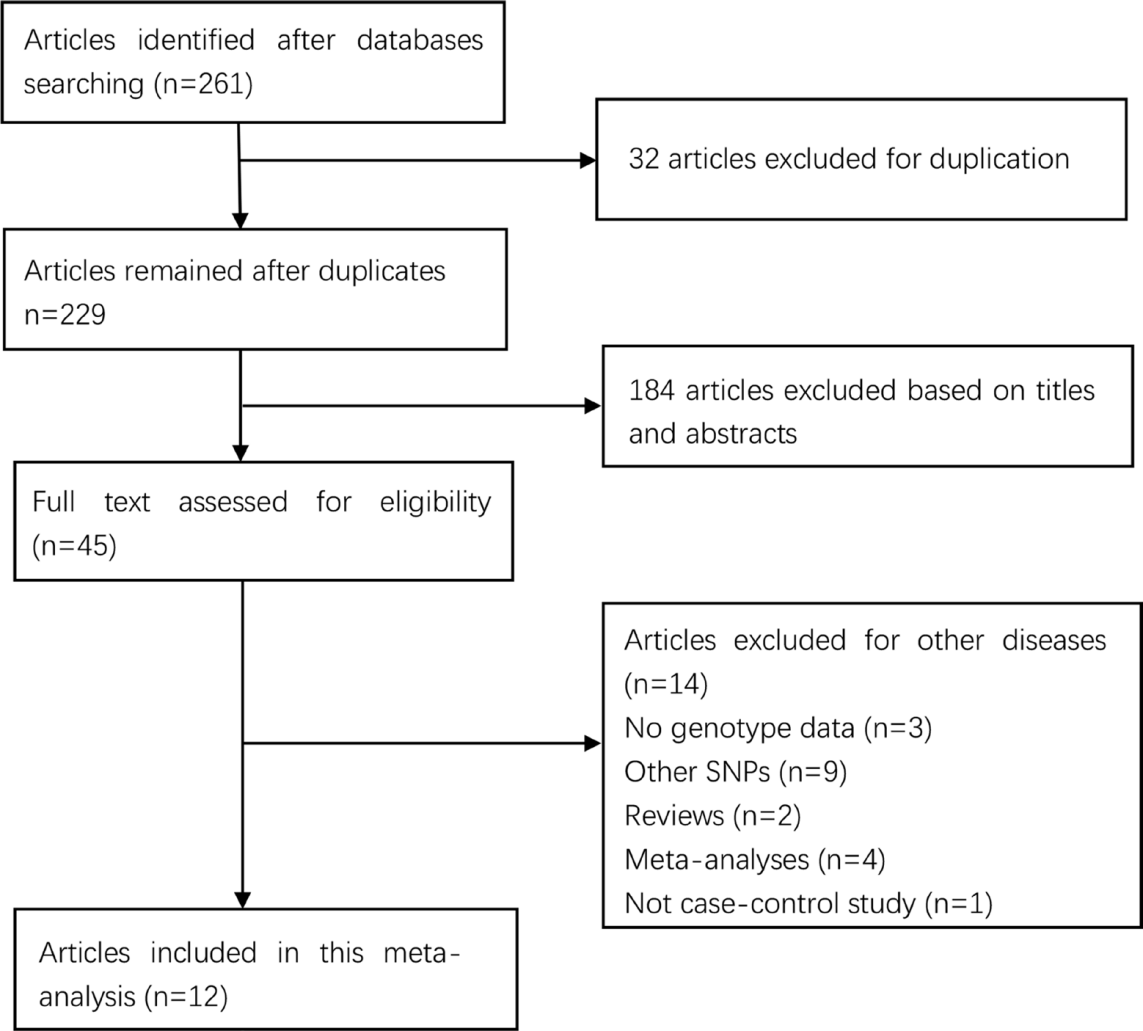
Selection for eligible citations included in this meta-analysis

261 articles were retrieved after initial search, and 45 full articles were identified for possible inclusion. 33 articles were excluded due to the following reasons: 9 investigated other polymorphisms; 3 did not provide detailed genotyping data; 14 studied coronary artery disease (CAD); 3 were reviews; 3 were meta-analyses and 1 was not case-control study. In total, 12 eligible studies involving 1,696 cases and 3,039 controls were included. Selection for eligible studies in this meta-analysis was presented in Figure 1. The detailed characteristics of all the selected studies were summarized in Table 1.

**Table 1:** Characteristics of included studies

Surname	Year	SOC	Nationality	Ethnicity	Number of cases			Number of controls			HWE	NOS
					AA	AG	GG	AA	AG	GG		
Nasibullin	2016	HB	Russian	Caucasian	101	152	62	90	145	51	Y	6
Gazi	2014	HB	Turkey	Caucasian	12	27	9	8	33	26	Y	7
Burazynska	2012	HB	Poland	Caucasian	69	44	5	272	379	173	Y	8
Liu	2011	HB	China	Asian	105	49	10	130	138	34	Y	6
Mohamed	2010	HB	Egypt	Caucasian	17	28	28	2	11	37	Y	7
Sakowicz	2010	PB	Poland	Caucasian	54	N/A	106*	48	69	14	Y	8
Aminian	2007	HB	Iran	Caucasian	42	77	33	36	69	35	Y	8
Podgoreanu	2006	HB	America	Caucasian	14	26	12	50	177	155	Y	8
Milutinovic	2006	HB	Slovenia	Caucasian	47	72	33	65	109	41	Y	6
Wei	2005	HB	China	Asian	76	35	7	101	103	26	Y	6
Wang	2005	HB	China	Asian	96	61	8	91	90	18	Y	7
Jiang	2002	PB	German	Caucasian	63	78	38	60	66	87	N	7

SOC, source of controls; PB, population-based controls; HB, hospital-based controls; NOS, Newcastle-Ottawa Scale; HWE: Hardy–Weinberg equilibrium.

*The Combined number of AG and GG genotypes; N/A: not available.

### Quantitative synthesis of data

**Figure 2:**
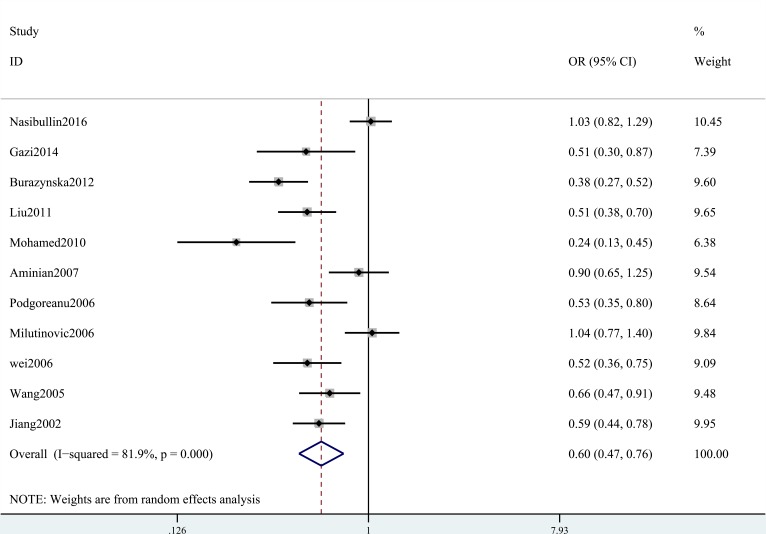
ORs and 95 % CIs from individual studies and pooled data for the association between ICAM-1 gene rs5948 polymorphism and MI in all subjects (G vs. A)

**Figure 3:**
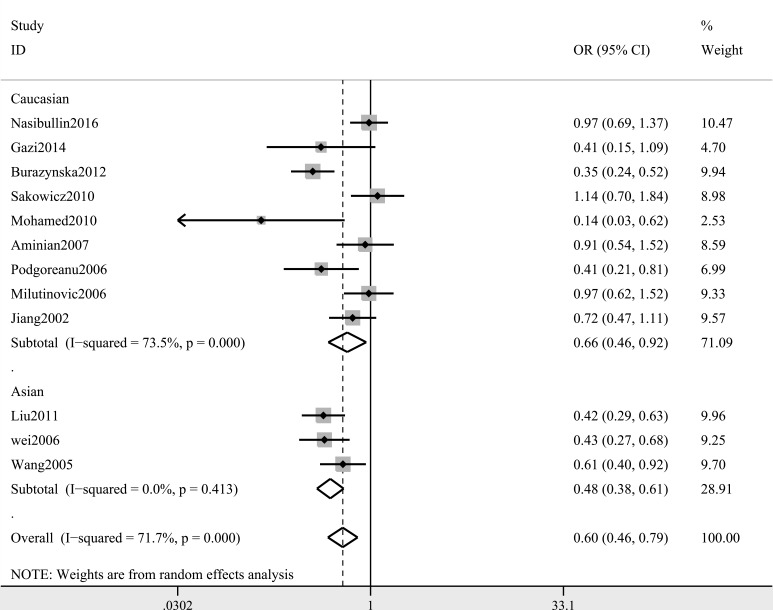
Stratification analysis of ethnicity shows odds ratio for the association between ICAM-1 gene rs5948 polymorphism and MI risk (AG+GG vs. AA)

**Figure 4:**
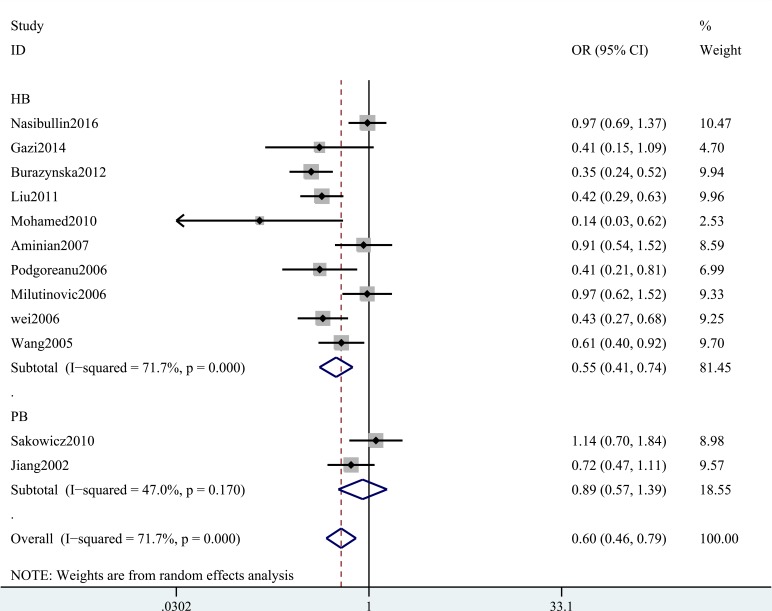
Stratification analysis of SOC shows odds ratio for the association between ICAM-1 gene rs5948 polymorphism and MI risk (AG+GG vs. AA) .

**Figure 5:**
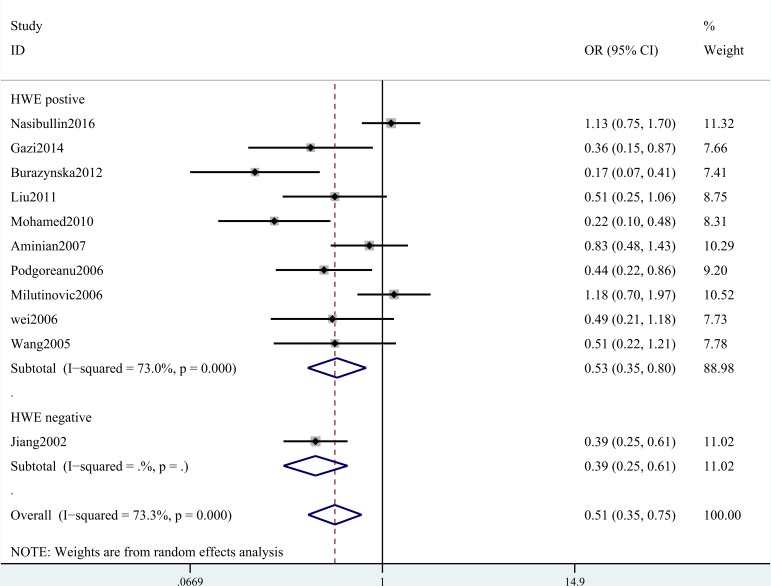
Stratification analysis of HWE status shows odds ratio for the association between ICAM-1 gene rs5948 polymorphism and MI risk (GG vs. AA+AG)

A summary of the meta-analysis findings of the associations between the ICAM-1 rs5948 polymorphism and MI is provided in Table 2 and Table 3. As shown in Table 2, ICAM-1 rs5948 polymorphism was associated with the decreased risk of MI (G vs. A: OR, 0.60; 95% CI, 0 0.47–0.76, *P* < 0.001; Figure 2). Stratification analyses were conducted according to ethnicity, source of controls (SOC) and Hardy-Weinberg Equilibrium (HWE) status. In Analysis after stratification by ethnicity, significant association between ICAM-1 rs5948 polymorphism and MI risk was observed not only among Caucasian populations (AG+GG vs. AA: OR = 0.66, 95 % CI 0.46–0.92, *P* < 0.001, Figure 3) but also among Asian populations (AG+GG vs. AA: OR = 0.48, 95 % CI 0.38–0.61, *P* < 0.001, Table 3). Stratification analysis of SOC found that ICAM-1 rs5948 polymorphism decreased the risk of MI among hospital-based controls (AG+GG vs. AA, Figure 4), but not among population-based controls (Table 3). One study did not conform to HWE, but stratification analysis of HWE status revealed the conclusions of the remaining studies did not significantly alter (GG vs. AA+AG, Figure 5), suggesting that the results of ICAM-1 rs5498 polymorphism were trustworthy.

**Table 2:** Meta-analysis of association between ICAM-1 rs5498 polymorphism and Myocardial infarction

Comparison	OR(95%CI)	*P*-value	P for heterogeneity	I^2^ (%)	Model
G vs. A	**0.60 (0.47,0.76)**	< 0.001	< 0.001	81.9	Random
AG+GG vs. AA	**0.60 (0.46,0.79)**	< 0.001	< 0.001	71.7	Random
GG vs. AA+AG	**0.51 (0.35,0.75)**	< 0.001	< 0.001	73.3	Random
GG vs. AA	**0.41 (0.26,0.65)**	< 0.001	< 0.001	75.0	Random
AG vs. AA	**0.66 (0.52,0.83)**	< 0.001	0.014	55.1	Random

*Bold values are statistically significant (*P* < 0.05).

**Table 3:** Summary of the subgroup analyses in this meta-analysis

Comparison	Category	Category	Studies	OR (95% CI)	*P*-value
G vs. A	Ethnicity	Caucasian	8	0.61 (0.44,0.84)	0.003
		Asian	3	0.56 (0.46,0.68)	< 0.001
	SOC	HB	10	0.60 (0.46,0.79)	< 0.001
		PB	1	0.59 (0.44,0.78)	< 0.001
	HWE	Yes	10	0.60 (0.46,0.79)	< 0.001
		No	1	0.59 (0.44,0.78)	< 0.001
AG+GG vs. AA	Ethnicity	Caucasian	9	0.66 (0.46,0.92)	0.016
		Asian	3	0.48 (0.38,0.61)	< 0.001
	SOC	HB	10	0.55 (0.41,0.74)	< 0.001
		PB	2	0.89 (0.57,1.39)	0.614
	HWE	Yes	11	0.55 (0.41,0.74)	< 0.001
		No	1	0.89 (0.57,1.39)	0.614
GG vs. AA+AG	Ethnicity	Caucasian	8	0.51 (0.31,0.82)	0.006
		Asian	3	0.51 (0.32,0.81)	0.005
	SOC	HB	10	0.53 (0.35,0.80)	0.002
		PB	1	0.39 (0.25,0.61)	< 0.001
	HWE	Yes	10	0.53 (0.35,0.80)	0.002
		No	1	0.39 (0.25,0.61)	< 0.001
GG vs. AA	Ethnicity	Caucasian	8	0.41 (0.23,0.75)	0.004
		Asian	3	0.38 (0.23,0.61)	< 0.001
	SOC	HB	10	0.40 (0.24,0.68)	0.001
		PB	1	0.42 (0.25,0.70)	0.001
	HWE	Yes	10	0.40 (0.24,0.68)	0.001
		No	1	0.42 (0.25,0.70)	0.001
AG vs. AA	Ethnicity	Caucasian	8	0.75 (0.57,1.00)	0.046
		Asian	3	0.64 (0.42,0.99)	< 0.001
	SOC	HB	10	0.62 (0.49,0.78)	< 0.001
		PB	1	1.13 (0.69,1.82)	0.631
	HWE	Yes	10	0.62 (0.49,0.78)	< 0.001
		No	1	1.13(0.69,1.82)	0.631

SOC, source of controls; PB, population-based controls; HB, hospital-based controls.

**Figure 6:**
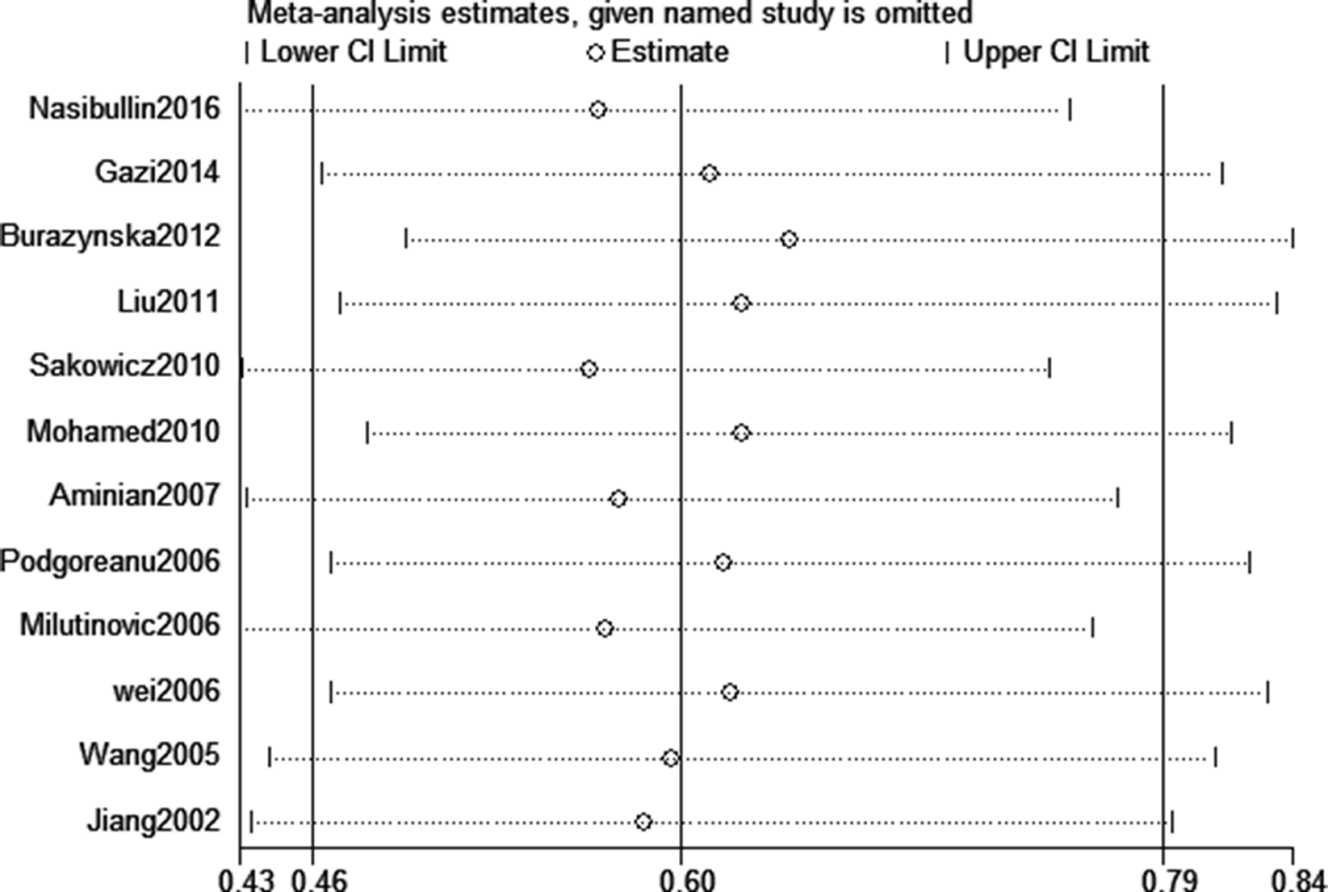
Sensitivity analysis for assessing the stability of the data of this meta-analysis (AG+GG vs. AA)

**Figure 7:**
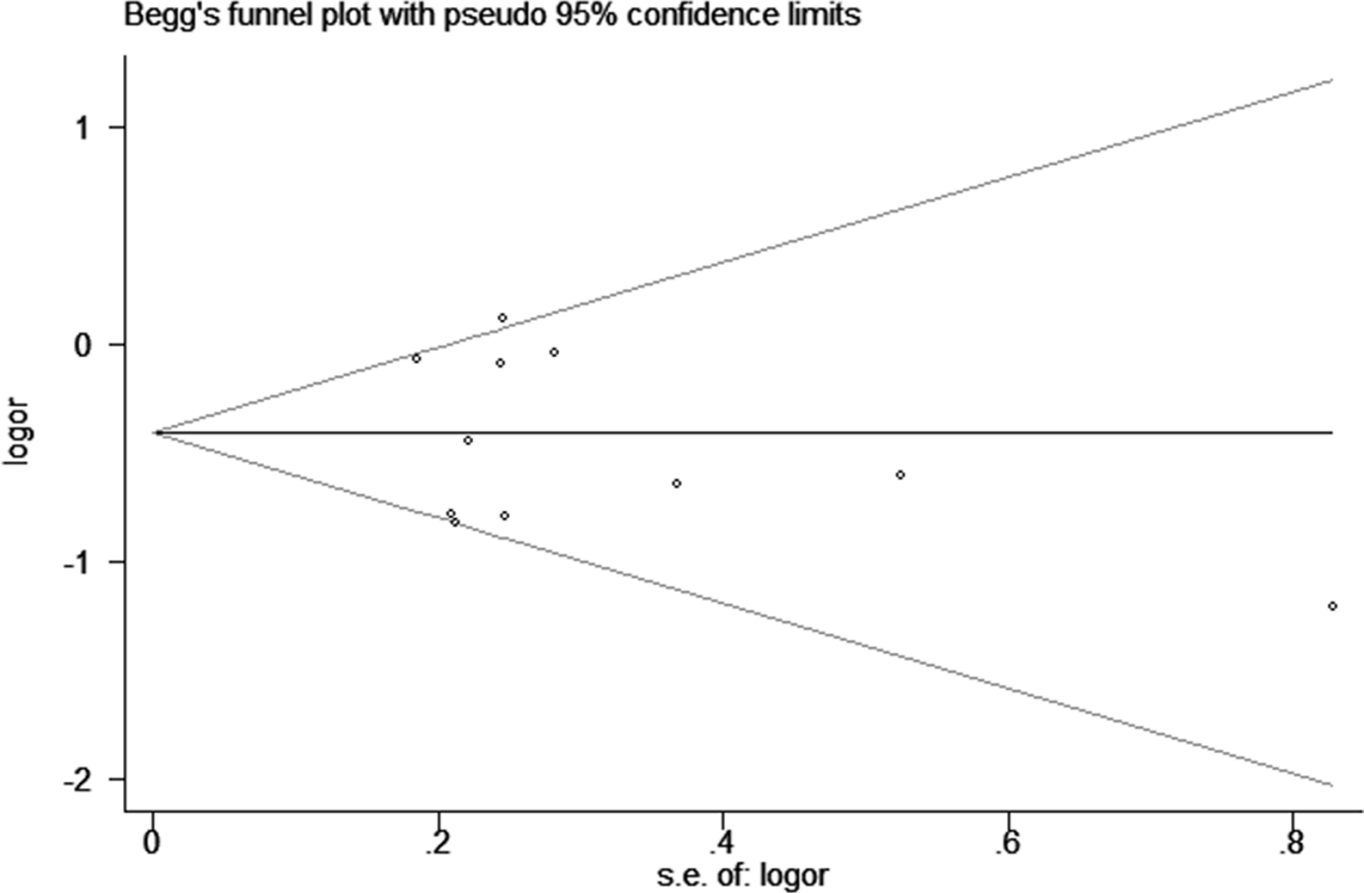
Begg's funnel plot of associated with ICAM-1 gene rs5948 polymorphism risk (AG vs. AA)

Sensitivity analysis was conducted to evaluate the effect of each study on the pooled ORs by omitting each study in turn. The pooled ORs were not affected by excluding any study (AG+GG vs. AA, Figure 6). Meanwhile, the potential publication bias (G vs. A, *P*_begg_ = 0.675 and *P*_egger_ = 0.548; AG+GG vs. AA, *P*_begg_ = 0.815 and *P*_egger_ = 0.593) was analyzed by performing both of the Begg's and Egger's tests (AG vs. AA, Figure 7). We found no obvious publication bias in this meta-analysis.

## DISCUSSION

In this current meta-analysis, ICAM-1 gene rs5498 polymorphism showed a significant association with the decreased risk of MI. Stratification analysis of ethnicity found that rs5498 polymorphism decreased the risk of MI among Caucasians and Asians.

ICAM-1 is a transmembrane glycoprotein in the immunoglobulin superfamily, which plays an important role in cell adhesion and signal transduction [23]. Ley *et al*. found that ICAM-1 is responsible for formation, growth and rupture of atheroma [24]. A circulating or soluble form of ICAM-1 with elevated levels were also observed in patients with atherosclerosis and heart failure [25]. A host of studies attached importance to assess the possibility that ICAM-1 gene polymorphism may become a new biomarker for MI [11–22]. Eight studies found that ICAM-1 rs5498 polymorphism was associated with a significantly increased risk of MI [12, 14, 16–18, 20–22]. However, the remaining studies failed to replicate this association [11, 13, 15, 19]. Therefore, we thought it was necessary to perform a meta-analysis to yield a more accurate conclusion on the association between ICAM-1 gene rs5498 polymorphism and MI risk.

Several meta-analyses investigated the association between ICAM-1 rs5498 polymorphism and CAD before [26–29]. They all indicated that rs5498 polymorphism was a risk factor for CAD [26–29]. Among these meta-analyses, only one study by Li *et al*. found that ICAM-1 rs5498 polymorphism could increase the risk of MI [26]. In this meta-analysis, several inevitable defects should be considered [26]. First, two studies without related genotype data were included in MI group [22, 30]. Second, they did not include a Slovenian study [15], which actually met the inclusion criteria. Third, they did not perform the subgroup analyses of ethnicity, SOC and HWE among MI groups. To the best of our knowledge, this study is the first meta-analysis to explore the association between ICAM-1 gene rs5498 polymorphism and MI risk. In this meta-analysis, we found rs5498 polymorphism was a protective factor for MI, which was different from the finding of Li *et al*. [26]. Stratification analysis of ethnicity also indicated that this polymorphism was associated with a decreased risk of MI among Caucasians and Asians. Obviously, this study did not obtain different results among different races. However, this meta-analysis only included Caucasian and Asian populations, but without other ethnicities such as Africans. Further studies among other ethnic populations are necessary to verify these findings. We thought our data were more robust than those of previous meta-analyses. More eligible studies were included in this meta-analysis [13, 15, 17]. In addition, the power analysis indicated that this meta-analysis had a power of 94.3% to detect the effect of rs5498 polymorphism on MI susceptibility with an OR of 0.60. Furthermore, subgroup analyses of ethnicity, SOC and HWE also revealed this SNP was associated with the increased risk of MI. Finally, sensitivity analysis indicated that no single study could significantly alter the pooled ORs, suggesting that our results were reliable.

The potential limitations should be taken into consideration. First, MI is a multifactorial disease and may be affected by several confounding factors, such as History of MI, gender, and age. Thus, the function of a single SNP is limited. Second, sample size was relatively small and high between-study heterogeneity was observed in several genetic models. Third, only Caucasian and Asian populations were included in this meta-analysis and the results may be not applied to other ethnic groups. Fourth, the sample size of this meta-analysis was not very large. Fifth, the subgroup analysis might have insufficient statistical power to assess the real association. Sixth, we performed this meta-analysis with crude ORs since studies included in this meta-analysis lacked sufficient data for adjustment for confounding factors, which might affect the stability of our results.

In summary, our meta-analysis confirms that ICAM-1 gene rs5498 polymorphism is a protective factor for MI. Stratified analysis of ethnicity indicates that ICAM-1 rs5498 polymorphism is associated with the decreased risk of MI among Caucasians and Asians. Further epidemiological studies with larger simple sizes from different geographic regions are needed to confirm our findings.

## MATERIALS AND METHODS

### Literature search

Two investigators carried out a systematic electronic search independently in PubMed, EMBASE and China Knowledge Resource Integrated Database to identify relevant studies using the following key words: “Intercellular Adhesion Molecule-1,” “ICAM-1,” “polymorphism,” “single nucleotide polymorphism,” “SNP” “Myocardial Infarction,” “Cardiovascular Stroke,” “Heart Attack,” “Myocardial Infarct”. No restrictions were placed on the search. Additional initially omitted studies (such as reference lists of identified studies) have been identified by hand screening.

### Inclusion and exclusion criteria

Eligible studies met the following inclusion criteria: (1) evaluating the relationship between ICAM-1 rs5498 polymorphism and risk of MI; (2) sufficient data for calculating the pooled odds ratio (ORs) with 95% confidence interval (CI); (3) case-control studies; (4) when studies had overlapping populations, the ones with the most complete data were included.

Exclusion criteria were as follows: 1) case-only studies; 2) meta-analysis or reviews; 3) studies that lacked detailed genotyping data; 4) duplicates of previous publications.

### Data extraction and quality assessment

Two authors reviewed and extracted data independently in accordance with the inclusion criteria. From each study, the following information was extracted: first author's surname, publication year, country of origin, ethnicity, and numbers of cases and controls. Authors were contacted to provide supplemental data, if data were not available in the eligible studies. When studies included subjects of more than one ethnicity, genotype data were extracted separately.

Two authors independently assessed the quality of the selected studies using the Newcastle-Ottawa Scales (NOS) [31]. Total NOS scores ranged from 0 to 9. A score ranging 5 to 9 stars is considered to be a generally high methodological quality while a score ranging 0 to 4 is regarded as a relatively poor quality. The discrepancies were resolved by discussion or consulting with a third author.

### Statistical analysis

All statistical analyses were performed using the Stata 11.0 software (StataCorp, College Station, TX, USA). Pooled ORs with corresponding 95% CIs were calculated to evaluate the strength of association between ICAM-1 rs5498 polymorphism and risk of MI. Stratification analyses were carried out by ethnicity, SOC and HWE. *P* < 0.05 was considered statistically significant. Taking possible between-study heterogeneity into consideration, we considered the presence of significant heterogeneity at the 10% level of significance and values of I^2^ exceeding 50% as an indicator of significant heterogeneity. When no heterogeneity was found with *P* > 0.10 or I^2^ < 50%, a fixed-effect model was used. Otherwise, a random-effects model was applied [32]. Sensitivity analysis was conducted to determine the effect on the test of heterogeneity and evaluate the stability of the results by omitting each study in turn. Genotype distributions in the controls were tested for confirmation of Hardy-Weinberg equilibrium using the χ2 test. Publication bias was evaluated by visual inspection of symmetry of Begg's funnel plot and assessment of Egger's test [33]; *P* < 0.05 was regarded as representative of statistical significance.

## Abbreviations

CVD: cardiovascular disease
CAD: coronary artery disease
MI: myocardial infarction
AMI: acute myocardial infarction
ICAM-1: intercellular adhesion molecule 1
SOC: source of controls
HWE: Hardy–Weinberg equilibrium
CI: confidence interval
OR: odds ratio
NOS: Newcastle- Ottawa Scale
SNP: single nucleotide polymorphism.
